# Defining the Global Research and Programmatic Agenda and Priority Actions for Voluntary Medical Male Circumcision for HIV Prevention

**DOI:** 10.1007/s11904-022-00640-y

**Published:** 2022-11-11

**Authors:** Megan E. Peck, Todd Lucas, Katherine S. Ong, Jonathan M. Grund, Stephanie Davis, Aisha Yansaneh, Valerian L. Kiggundu, Anne G. Thomas, Kelly Curran, Catharine Laube, Maaya Sundaram, Wole Ameyan, Lycias Zembe, Carlos Toledo

**Affiliations:** 1 Division of Global HIV and Tuberculosis, US Centers for Disease Control and Prevention, 3 Corporate Blvd NE, US1-1, Atlanta, GA 30329 USA; 2 Division of Global HIV and Tuberculosis, Centers for Disease Control and Prevention, Pretoria, South Africa; 3 Tucson, USA; 4 Office of HIV/AIDS, United States Agency for International Development (USAID), Washington, DC USA; 5 HIV/AIDS Prevention Program U.S. Department of Defense, San Diego, CA USA; 6 Jhpiego, Baltimore, MD USA; 7 Global Development Program, Bill & Melinda Gates Foundation, Seattle, WA USA; 8 Department of HIV, Viral Hepatitis and STIs, World Health Organization, Geneva, Switzerland; 9 United Nations Joint Programme on HIV/AIDS, Geneva, Switzerland

**Keywords:** HIV prevention, Voluntary medical male circumcision, HIV/AIDS, Sub-Saharan Africa

## Abstract

**Purpose of Review:**

Since 2007, voluntary medical male circumcision (VMMC) programs have been associated with substantially reduced HIV incidence across 15 prioritized countries in Eastern and Southern Africa. Drawing on the programmatic experience of global VMMC leaders, this report reviews progress made in the first 15 years of the program, describes programmatic and research gaps, and presents considerations to maximize the impact of VMMC.

**Recent Findings:**

Overall, key programmatic and research gaps include a lack of robust male circumcision coverage estimates due to limitations to the data and a lack of standardized approaches across programs; challenges enhancing VMMC uptake include difficulties reaching populations at higher risk for HIV infection and men 30 years and older; limitations to program and procedural quality and safety including variations in approaches used by programs; and lastly, sustainability with limited evidence-based practices. Considerations to address these gaps include the need for global guidance on estimating coverage, conducting additional research on specific sub-populations to improve VMMC uptake, implementation of responsive and comprehensive approaches to adverse event surveillance, and diversifying financing streams to progress towards sustainability.

**Summary:**

This report’s findings may help establish a global VMMC research and programmatic agenda to inform policy, research, and capacity-building activities at the national and global levels.

## Introduction

Voluntary medical male circumcision (VMMC) was first formally recommended in 2007 by the World Health Organization (WHO) and the Joint United Nations Programme on HIV/AIDS (UNAIDS) as an effective and essential HIV prevention strategy [1]. Since this recommendation, important advances have been made by VMMC programs in prioritized countries in Eastern and Southern Africa [2, 3]. These advances offer lessons learned, helping to identify opportunities for future programmatic growth. Drawing on the programmatic experience and expertise of global VMMC stakeholders, this report presents the progress made since 2007, programmatic gaps, and potential responses needed for VMMC to maximize its contribution to the 2030 targets to end the AIDS epidemic. The suggestions for considerations represent the shared opinion of authors leading the global VMMC portfolios of major funding agencies and organizations and international implementing agencies. The two questions this report seeks to answer are, first, what are the remaining research and programmatic gaps, and, second, what additional efforts may be considered to address these needs.

## Background

**Fig. 1:**
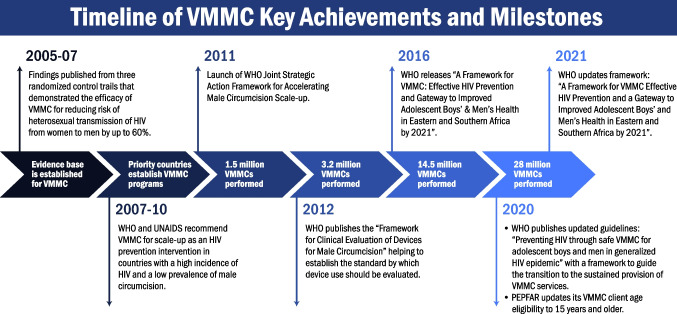
Timeline of VMMC key achievements and milestones

In 2007, WHO and UNAIDS’s recognition of VMMC as an efficacious prevention strategy was prompted by findings from randomized controlled trials (RCTs) conducted in Kenya, Uganda, and South Africa, demonstrating a reduction in the acquisition of HIV in men by up to 60% (Fig. 1) [4–7]. Countries with generalized HIV epidemics and low national or subnational male circumcision prevalence were prioritized following this global guidance. Countries originally prioritized included Botswana, Eswatini, Ethiopia, Kenya, Lesotho, Malawi, Mozambique, Namibia, Rwanda, South Africa, United Republic of Tanzania, Uganda, Zambia, and Zimbabwe, with South Sudan establishing a program in 2018 [8•]. The VMMC programs were country-led and incorporated into national HIV prevention portfolios with funding support primarily provided by the U.S. President’s Emergency Plan for AIDS Relief (PEPFAR) and national governments [2, 9]. It was recommended VMMC be offered as part of a package of services including the surgical removal of the foreskin, voluntary HIV testing, links to HIV care and treatment, HIV risk reduction education, condom provision, and referrals for screenings or treatment when patients are diagnosed with other sexually transmitted infections (STIs) [1, 10].

In 2011, WHO and UNAIDS published a framework to guide the development of VMMC programs [11]. The framework described two phases of the program and presented global targets to maximize the impact on HIV incidence. The first phase was initially intended to conclude by 2016 with circumcision coverage of at least 80% among 15–49-year-old males [2]. The expectation was once the first phase was completed; the high coverage achieved among older males would mean high-volume VMMC services would no longer be needed for these clients. However, the initial phase of the program continued beyond the intended timeline largely due to the policy and programmatic challenges of establishing and scaling up an elective surgical public health intervention and changes to the global male circumcision coverage targets based on updated modeling. By the end of 2010, the original 14 prioritized countries had all established national programs, and between 2007 and 2014, over 9 million VMMCs were performed [12]. The program experienced performance decreases of nearly 9% in 2015 and 2016, primarily due to reductions in global funding. However, in 2017, there was an increase to 4.0 million VMMCs performed compared to 2.8 million in 2016 due to an increase in PEPFAR funding [13]. The annual growth continued until 2020 when programs experienced a reduction in the number of VMMCs performed largely due to the COVID-19 pandemic. From 2007 to 2020, 26.8 million VMMCs were performed [14, 15].

From 2007 to 2020, adolescents aged 10–14 years comprised the greatest proportion of VMMC clients. In 2020, there was a shift in the age distribution of VMMC clients due to updated global guidance and changes in PEPFAR’s age eligibility restricting procedures to clients 15 years and older [16••, 17••]. PEPFAR made this policy decision following a data review showing a higher rate of adverse events (AEs) associated with VMMC in clients 10–14 years of age. Thus, despite its success, the scale-up phase did not follow the original projections for the program, given younger adolescents represented a disproportionally greater client volume than was expected in most countries, and uptake was somewhat stagnant among men 30 years and older [18].

After the initial scale-up phase, programs were meant to enter a second phase, the sustainability phase, where the focus would shift to younger adolescents and infants who were not eligible for VMMC during the first phase. Programs were intended to enter this phase once they had achieved at least 80% male circumcision coverage among 15–49-year-old males. Each country established a national program providing VMMC services to all infants up to 2 months old. During the implementation of this second phase, VMMC services were also meant to be integrated into national health systems. In practice, both proposed phases of the program were implemented differently from the original plan.

Although VMMC programs were implemented differently than initially planned, the growth and achievements of the VMMC program to date carry immediate and future benefits [19]. By 2020, the nearly 27 million VMMCs performed were estimated to have averted 340,000 new HIV infections [20]. The impact of medical male circumcision is estimated to be significantly more substantial on HIV incidence than non-medical circumcision. Unlike medical circumcision which is a standardized surgical procedure, the amount of foreskin removed during non-medical circumcision is not uniform, and the protective effects for HIV and STI prevention vary [21, 22]. Although VMMC programs have focused primarily on circumcision to prevent HIV among males, there is evidence demonstrating reductions in STI transmission in both men and women and reductions in cervical cancer in women [23, 24•, 25•]. To ensure VMMC programs continue to contribute to population-level benefits, it is important to understand the research and programmatic gaps hindering the progress of the program and the opportunities to progress.

## Programmatic and Research Gaps and Priority Actions

This section outlines relevant VMMC programmatic and research gaps and recommends priority steps to address them (Table 1). Identifying and responding to these challenges is important considering some programs are reaching high VMMC coverage requiring an updated programmatic focus and technical support.

**Table 1:** VMMC programmatic and research gaps and recommended priority actions for prioritized countries

VMMC program area	Programmatic and research gaps	Recommended priority actions
Estimating male circumcision coverage	National programmatic planning to reach HIV epidemic control benchmarks remains a challenge, particularly in areas with dynamic or limited denominator data	Global guidance for standardized data inputs and statistical processes to measure male circumcision coverage
	Robust estimates of VMMC coverage, particularly for subnational areas, are limited by the data currently available	Country-level strategies to ensure high-quality data (timely, accurate, and complete) to provide robust data inputs
		Standardized male circumcision module for country-level adaption in surveys, particularly to improve reporting of type of circumcision (medical or non-medical)
		When possible, adapt study design and statistical power of household surveys to improve measurement of male circumcision coverage
	Country-level support for establishing programmatic strategies to support long-term and sustainable efforts to estimate high coverage of VMMC	Global guidance and adaptable frameworks for identifying male circumcision coverage gaps among sub-populations with lower coverage
Enhancing uptake	Low uptake among specific groups of men who have lower circumcision coverage and are at higher risk for HIV, particularly among specific occupational groups such as fisherfolk, truck drivers, miners, and seasonal workers	Greater research on behavioral drivers of uptake, including use of compensation or incentives and when programs should use them
		Investigation of generalizability or consistency of factors that account for segmentation of VMMC clients
	Low uptake among older age groups (≥ 30 years) and lack of specialized approaches to target key groups	Leverage mapping and analytic technology, such as geographic information systems, to optimize use of resources and service provision
		Demand creation activities that communicate the benefits of VMMC to individuals and partners to address behavioral and interpersonal barriers
		Examine the cost/benefit of targeting clients 30 years and older
	Innovative technological limits due to gaps in specialized knowledge, infrastructure, and financial resources for demand creation	Digital and social media demand creation packages that can be adapted to meet the needs of each VMMC prioritized country
	Gaps in research to better understand the use of device-based male circumcision including ShangRing© compared to conventional surgical approaches	Understand preferences for type of male medical circumcision procedure offered, and the impact of device availability on VMMC uptake
Quality and safety	Lack of a standardized approach to appropriately manage certain severe adverse events	Establish a network to share safety and adverse event experiences across VMMC programs to improve outcomes
		Develop a responsive, standardized, and comprehensive approach to adverse event surveillance
		Categorization of attributes of clients at higher risk for AEs or with compliance challenges to improve and potentially prevent adverse outcomes
	Lack of standardized communication of benefits and risks of the procedure to aid clients’ decision-making process and lack of specific guidance on how best to assess capacity for informed consent/assent, particularly for younger age bands (≤ 19 years)	Create a comprehensive and easily understandable communication guide on the benefits and risk (i.e., standardize summaries) to strengthen client’s decision-making abilities during the informed consent process
		Global guidance to improve assessment of readiness of younger clients (≤ 19 years) and parents/guardian to comprehend benefits and risks of VMMC
	Lack of focus on the quality and safety of program areas beyond the procedure, such as sexual health, HIV testing services, demand creation, education, and communication for the client	Review and improve materials, standard operating procedures, and checklists for client counseling on sexual health, HIV testing services, referrals, etc
Sustainability	Each service delivery and strategic planning component needed to achieve sustainability for VMMC programs remains relatively unknown	Strategic inclusion of WHO health systems six building blocks and components framework for sustainable VMMC services into national planning (Table 2)
	Limited evidence-based sustainability practices to inform planning for sustainability	Measuring country progression towards sustainability is critical for countries to understand and assess their transition and identify needed support as well as best practices. Compiling country efforts towards sustainability will help to establish an evidence base and provide lessons learned
	Limited understanding of long-term workforce needs to sustain VMMC services	Engaging ministries of health and finance, in addition to policymakers, for VMMC integration and long-term workforce planning into national health priorities
	Lack of diversified funding and need for commitment to non-donor supported funding	Diversified financing schemes that include both domestic and external funding and greater resource mobilization through engagement of ministries of health and finance

### Estimating Male Circumcision Coverage

Circumcision coverage estimates play a critical role in VMMC program planning, target setting, and monitoring progress towards HIV epidemic control goals. The VMMC programs use a variety of survey and sampling methodologies, statistical tools, and data to estimate coverage. National household surveys, including the Population-based HIV Impact Assessment (PHIA) and the Demographic and Health Surveys (DHS), are commonly used to inform the estimation of VMMC coverage [21, 26, 27••]. However, evidence suggests male circumcision coverage estimates are unreliable with inconsistencies between data sources, coverage continuously exceeding 100% in some areas, and continued high demand in areas with high estimated coverage [28].

There are limitations to national household surveys when used to estimate coverage. Typically, the subnational circumcision estimates are less robust than national-level estimates when using national household surveys. Less robust estimates at the subnational level are partly because of inaccurate or dynamic denominator data with limited information on internal and external population migration, particularly in countries with older census data, high mobility, or areas with an unstable population with a high proportion of unregistered immigrants, migrant workers, and refugees. This denominator issue is particularly pronounced in countries where granular clinical data are missing at lower administrative levels and in countries with great variation in coverage across geographic regions. The lack of robust subnational-level estimates is partly because national household surveys are not designed statistically for estimating male circumcision coverage.

Additionally, there are limitations to how male circumcision is measured, usually through self-report, with challenges distinguishing medical and non-medical male circumcision. Previous research has found conflicting findings with self-reported circumcision status may be subject to reporting errors in some settings. At the same time, in other areas, there is high concurrence with physical exams [29, 30]. These inconsistencies can lead to over- and underestimating male circumcision coverage and an overall lack of robust and comparable estimates.

According to anecdotal reports, another challenge to estimating accurate coverage is VMMC may be “replacing” non-medical male circumcision in some settings. However, the models and tools used to estimate coverage sometimes assume the proportion of non-medical male circumcision in a national population remains constant. Therefore, additional research is needed to estimate non-medical male circumcision coverage more precisely in various settings.

Robust estimation of male circumcision coverage is important for all aspects of the program but is particularly critical for setting program targets. UNAIDS establishes VMMC targets roughly every 5 years, aligning with goals for ending the AIDS epidemic. Prioritized countries adopt these targets nationally, typically establishing 5-year plans to reach these goals. In addition, PEPFAR supports national governments in establishing program targets annually. However, the accuracy of these targets, particularly at the subnational level, is limited by the reliability of male circumcision coverage estimates discussed above. Overall, there has also been a lack of a standardized approach to establishing coverage estimates and set targets. In 2020, the UNAIDS Reference Group on Estimates, Modeling, and Projections, along with partners, worked with prioritized countries to integrate VMMC into the UNAIDS HIV estimates process to standardize the process [31, 32]. However, official global guidance for standardized estimation of circumcision coverage is needed to enable routine monitoring and more accurate and precise male circumcision coverage estimates.

Defining the types of data collected to supplement national household surveys could help countries determine when additional survey data is needed and which sources are best used in triangulating data. Guidance may be helpful to identify programmatic and service delivery gaps among specific eligible sub-populations with lower male circumcision coverage. Establishing standardized framework countries could easily adapt for estimating coverage at the district or lower administrative level or help identify pockets of low coverage could increase reliability in estimates. Additionally, improvements can be made to national household surveys to improve the accuracy of VMMC reporting. For example, a standardized male circumcision module could be included in national household surveys to more accurately document circumcision status among males through direct observation of circumcision status. As countries reach higher male circumcision coverage, estimating VMMC coverage will become increasingly challenging considering, as coverage rises, estimates become increasingly sensitive to errors in target population estimates.

### Enhancing Uptake

Despite the success of the VMMC program, there is an ongoing effort to improve strategies to increase VMMC uptake to reduce HIV incidence to reach epidemic control. Programs currently use a variety of approaches and strategies to tailor the most successful interventions for enhancing uptake [17••, 33–35]. However, key gaps remain in understanding factors accounting for the choice to undergo the procedure, including motivators, barriers, and experiences [31, 36]. Programs have reported challenges reaching specific groups of men, some of whom are at higher risk of HIV infection and hard to reach for services. Hard-to-reach groups commonly include mobile workers, fishermen, truck drivers, miners, and seasonal workers [17••, 37•]. Populations at higher risk such as men diagnosed with STIs, male partners of female sex workers, and serodiscordant partnerships can also pose specific demand creation challenges [38, 39]. Another important programmatic challenge is the limited voluntary uptake of circumcision among males 30 years and older who are more likely to be married or cohabiting, finding it challenging to justify VMMC to their partners [40].

Programs are also facing a digital divide. There is varying capacity to implement technology in social media campaigns across prioritized countries. Only programs with the specialized knowledge, infrastructure, and financial resources to develop the mobile tools have the advantage of utilizing the algorithms social media affords reaching targeted groups with tailored messaging. Programs have experienced recent success in using innovative technology to target specific audiences through social media, particularly to engage older men [41].

A promising strategy for improving uptake of VMMC is psychographic-behavioral segmentation, using human-centered design (HCD). Through segmentation, potential VMMC clients are categorized according to certain characteristics, which allows for specific messaging based on a client’s profile [42]. Initial studies found differing client characteristics in Zimbabwe and Zambia, indicating the importance of tailored interventions [43, 44]. Another important factor for increasing uptake is understanding the stage of change in which the client is in when they are approached for VMMC [45]. Additional evidence is needed to understand how readiness for the procedure relates to the uptake of services. In addition to psychographic approaches, demographic and geographic segmentation are important for developing a strategic approach to demand generation. Demographic segmentation may be useful for increasing uptake among older age groups, because demand creation approaches are specifically tailored to address their needs. Another demographic segmentation factor for consideration is a client’s financial status. Wage loss from time off work for recovery after VMMC, including the perceived time needed, has differential effects on men depending on their financial status. More information is needed to understand situations in which compensation or incentives are most sensible for programs to offer and leverage proven approaches to reach different sub-demands. Geographic segmentation by clustering clients according to their location could benefit demand creation planning. Mapping and analytic technology such as geographic information systems can help programs plan for optimal use of resources and guide services with the greatest potential for VMMC conversion.

Device-based male circumcision may influence men’s perceptions of VMMC and may be a promising approach to increase uptake [17••, 46]. However, gaps still exist on whether device-based male circumcision increases demand among those previously hesitant or merely replaces surgical VMMC among those who eventually would have sought services. Research is also needed on the cost/benefit of targeting older men (≥ 30 years) for VMMC when fewer resources are believed to be required for demand generation targeting younger age groups (≤ 19 years) [47]. Once a program has reached high coverage and shifted towards sustainability, strategies to enhance uptake could focus on males aging into eligibility [48] with ongoing evaluations to ensure strategies continue to be effective on newly eligible clients.

### Quality and Safety

Voluntary medical male circumcision is a preventive procedure typically performed on healthy males. Safety has been a critical priority for VMMC programs, and VMMC has demonstrated it is a safe procedure. Adverse events have been monitored since program inception, and overall, < 2% of VMMC clients have been identified through passive reporting as having moderate or severe AEs [49, 50]. In addition, national VMMC programs have made critical adjustments in response to safety signals, such as implementing mitigation measures in response to tetanus cases associated with VMMC, halting the use of forceps-guided circumcisions in clients 10–14 years old, enhancing pre-circumcision screening for bleeding disorders, and raising client age eligibility for conventional surgical VMMC to 15 years and older due to an increase in specific adverse events reported in 10–14-year-old clients [51–53].

Quality assurance and quality improvement activities commonly practiced by VMMC programs include AE surveillance, AE investigations and contributing root cause analyses, external quality assessments, and continuous quality improvement. However, each national program has its approach to quality and safety, which often changes over time and is frequently determined in reaction to an event. Therefore, the lack of a deliberate approach to program quality can lead to inadequate monitoring, missed epidemiologic signals, insufficient AE investigations and analyses, improper corrective actions, and ultimately, missed opportunities to improve safety for clients and staff.

With any surgical intervention, it is impossible to eliminate all complications. When AEs occur, timely diagnosis and optimal management can minimize long-term sequelae. Although tools and guidance have been developed to standardize and improve the management and prevention of commonly reported AEs, there is considerable variation in how national VMMC programs manage certain severe AEs [48, 54, 55]. Some programs have a well-planned AE management system, including designated specialty care providers or centers of excellence. Still, many lack a standardized approach or local capacity to manage these cases appropriately.

Informed consent/assent is a critical component of preoperative counseling. Counseling on risks and benefits can be transparent and delivered in easy-to-understand language with ample opportunities for clients to ask questions. Clients, especially younger adolescents, have a wide range of maturity and capacity to understand the procedure, potentially impacting their ability to give informed consent/assent. Although the general need for client understanding of risks and benefits prior to surgery is well-accepted, there is a potential need for greater assessment and support of adolescents’ capacity for autonomous decision-making in healthcare settings [56].

Improving VMMC safety is a constant priority for VMMC program planners and implementers. Recommended priority steps for improving the quality and safety of VMMC programs include a responsive, standardized, and comprehensive approach to AE surveillance. Through continuous quality improvement, surveillance data for action gives purpose to surveillance by translating safety events to improved outcomes for future clients and overall improved program safety. Continued monitoring of quality and safety activities can remain a critical component of VMMC programs. Standardized summaries of risks and benefits could be developed for VMMC programs to translate and use for eligible clients to improve informed consenting practices. In addition, global guidance is needed on how VMMC counselors can assess the ability of younger clients and their parents or guardians to comprehend and provide consent or assent for VMMC.

Robust networks and coordination across programs are needed to improve the safety and quality of VMMC programs. Establishing a network of shared safety experiences within and across national VMMC programs could amplify best practices and provide a mechanism to share AE management experiences. Additionally, improved coordination within and among countries to optimize the management of serious, complex AEs is needed. Additional research on the categorization of attributes of clients at higher risk for AEs or who missed follow-up appointments could help to identify higher risk clients to prevent adverse outcomes. Additionally, male circumcision devices warrant monitoring to inform programming decisions around safety and sustainability. Finally, another important gap is that the program has primarily concentrated on the quality of the procedure but has not focused as rigorously on the quality of other areas such as HIV testing services, medical record keeping, demand creation, and client education. Additional attention and resources may be needed to ensure all aspects of quality for the VMMC program are rigorously monitored.

### Sustainability

Following the first 15 years of program expansion, the sustainability of national VMMC programs is becoming increasingly important. Although sustainability was initially conceived as a distinct phase of program transition, sustainability of VMMC will require a more gradual evolution including measured integration of VMMC services into national health systems. The sustainability of VMMC is particularly important with economic modeling predicting the program will continue to be a cost-effective prevention strategy and, in fact, cost-saving in most countries over a 50-year horizon [57]. In 2020, as part of the updated recommendations for VMMC, WHO published a framework for sustaining VMMC services [17••]. The framework builds upon the WHO’s health system “building blocks” and integrates VMMC-specific information [58]. The sustainability of VMMC is defined in the following ways: (1) the capacity of VMMC services to maintain high male circumcision coverage (≥ 90%); (2) the integration of VMMC services into a country’s health system; (3) strong country ownership and leadership; and (4) resource mobilization may include domestic and external funding. VMMC sustainability is a new area for national programs, and programmatic and research gaps remain, and each strategic planning component for sustainable VMMC services remains relatively unknown. One area which poses a challenge is long-term workforce needs. Although the capacity building of the health workforce, particularly nurses, to perform the surgical procedure has been a major success of VMMC implementers and donors, the long-term resource and policy could sustain this workforce capacity remain unknown.

Priority steps could be taken to progress successfully to fully sustainable VMMC programs. The VMMCs programs have relied largely on donor support, and the need for diversified financing streams is evident, as donor-funded programs alone are not sustainable. Engaging ministries of health and finance will be critical as national health priorities are financed. However, careful evolution of financial responsibility is essential, with most countries lacking the resources to fully support HIV prevention services. Priority components for VMMC sustainability for each of the building blocks are outlined in the WHO framework summarized in Table 2. Key steps for the first building block of finance include estimating country resource needs and inclusion into national health plans. Inclusion of VMMC programs into national health plans could include integration with other health services such as national sexual and reproduction health programs for adolescents and males. For health workforce planning, the establishment of systems to monitor staff retention and integration of the VMMC workforce into national workforce planning can be considered. Recommended initial steps towards workforce sustainability include the incorporation of VMMC into pre-service health care worker training programs [59]. Recommended steps for the strategic information building block include integrating data collection into routine monitoring and evaluation systems and modernizing data collection.

**Table 2:** WHO health systems six building blocks and components framework for suitable VMMC services

Building block	Component
Finance	• Resource allocation and mobilization • Purchasing of services • Financial risk protection
Health workforce	• Health workforce planning • Pre-service and continuing education • Management, supply, and supervision
Strategic information	• Data collection and management • Data quality • Data analysis and use • Safety monitoring
Supplies and equipment	• Norms and standards • Procurement, supply, and distribution • Quality of VMMC supplies and equipment
Leadership and governance	• Program leadership and coordination • Accountability, oversight, and regulation • Inter-sectoral coordination • Health sector plans and policies
Service delivery	• Access (strategic planning of health services) • Reorienting service delivery models • Empowering and engaging people • Safety and quality

Note: Adapted from WHO Preventing HIV Through Safe Voluntary Medical Male Circumcision for Adolescent Boys and Men in Generalized HIV Epidemics. 2020. Available at: https://www.who.int/publications/i/item/978-92-4-000854-0

For the supplies and equipment building block, integration of procurement and distribution of supplies into national procurement systems are recommended. This area may be easier to transition, with several ministries of health already procuring supplies for other health services. Although the scale-up phase of the program primarily relied on single-use instruments, more recent guidance has prioritized reusable instruments, not only for the sustainability of services but also to reduce costs and environmental impact. Considerations for the leadership and governance building block focus on integration with local governance. Leadership and governance for VMMC have been country-led since the program’s inception and maintaining this momentum will be critical for the program’s sustainability. Lastly, key actions for the service delivery building block focus on strategic planning of health services and promotion of VMMC as part of a comprehensive package of care.

Measuring a country’s progress towards sustainability is critical for countries to understand and assess their transition. A 2022 landscape analysis conducted by WHO and UNAIDS applied the above-referenced framework and summarized the status of each country’s VMMC program in relation to the sustainability of services. The overwhelming majority of the WHO building block designations were either at an early or intermediate phase with minimal or only a few sustainable features present. A full report on the analysis is expected to be published in mid-2022.

Many countries have taken steps towards sustainability. Actions range from developing national plans for sustainability to piloting the transition of service delivery from donor-funded community organizations to government services [60, 61]. For example, several countries have tried to incorporate early infant male circumcision (EIMC) as part of their sustainability plan, but no national EIMC programs are currently active. In 2019, PEPFAR support for all EIMC activities was ceased after a careful review of impact and safety data. In 2020, WHO further cautioned countries to carefully consider the risks and benefits, human rights, and resources to safely deliver EIMC services for HIV prevention [17••].

## Conclusions

The issues presented in this report contribute to policy and research agenda-setting and action at the global and national levels. Continuing the progress VMMC programs have made during the first 15 years of the program is important to reduce HIV incidence and achieve the UNAIDS 2025 AIDS Targets for 90% of males 15 years and older to have access to VMMC in prioritized countries [62–64]. However, important VMMC programmatic challenges remain requiring budgetary commitments, leadership, and commitment across governments and global partners. Programs have demonstrated their ability to grow and adapt and will continue to do so as they work towards addressing programmatic and research gaps.
